# Investigating barriers to clinical academic careers amongst undergraduate medical students

**DOI:** 10.1016/j.clinme.2026.100657

**Published:** 2026-10-18

**Authors:** Ranya V. Kumar, Caleb Cole, Jacob Vincent, Prakrit R. Kumar, Nicholas R. Evans

**Affiliations:** a School of Clinical Medicine, University of Cambridge, Cambridge, United Kingdom; b Department of Trauma and Orthopaedics Surgery, University Hospital Coventry & Warwickshire, Clifford Bridge Rd, Coventry, United Kingdom; c Warwick Medical School, University of Warwick, Coventry, United Kingdom; d Department of Clinical Neurosciences, University of Cambridge, Cambridge, United Kingdom

**Keywords:** Medical education, Research, Academic medicine, Undergraduate

## Abstract

**Introduction:**

There is a critical shortage of academic clinicians in the UK, particularly amongst minority ethnic groups. This study aimed to identify students’ perceived barriers to engagement in research, and evaluate whether demographic characteristics influenced these perceptions, in order to identify opportunities to improve training and promote clinical academic careers.

**Methods:**

A 59-item structured survey was distributed between November 2023 and January 2024 to medical students at the University of Cambridge. It incorporated 45 questions using a five-point Likert scale to measure knowledge of research types, confidence with research skills, and understanding of academic careers, producing a total (global) score of 45–225.

**Results:**

One hundred and twenty-six students responded. One in four students reported a poor understanding of the clinical academic career pathway. Few students felt confident performing core research steps, such as designing a study (n = 23, 18.3%), analysing data (n = 27, 21.4%), and writing it as a full research manuscript (n = 16, 12.7%). On multivariable analysis, Caucasian ethnicity and year of study were independently associated with increased global scores (betas of 26.4 and 12.5 respectively, both p < 0.05), but not sex (beta −0.92, p = 0.88). Consequently, students from non-Caucasian backgrounds reported 11.8% less confidence in their understanding of research and clinical academic careers.

**Conclusion:**

Students perceive that insufficient understanding, skills and opportunities inhibit their engagement in research. These feelings are heightened in those at early stages of their training and those of non-Caucasian ethnicity. Thus, there is a need for dedicated training delivered early in medical training to increase representation and inclusivity in clinical academia.

## Highlights

• Medical students demonstrate low levels of research experience and awareness of clinical academic career structures.
• They perceive poor understanding, skills & opportunities as barriers to research.
• These barriers are felt more strongly in those of non-Caucasian ethnicity.
• There is demand amongst students for dedicated, standardised training in research.

## Graphical Abstract

**Figure:**
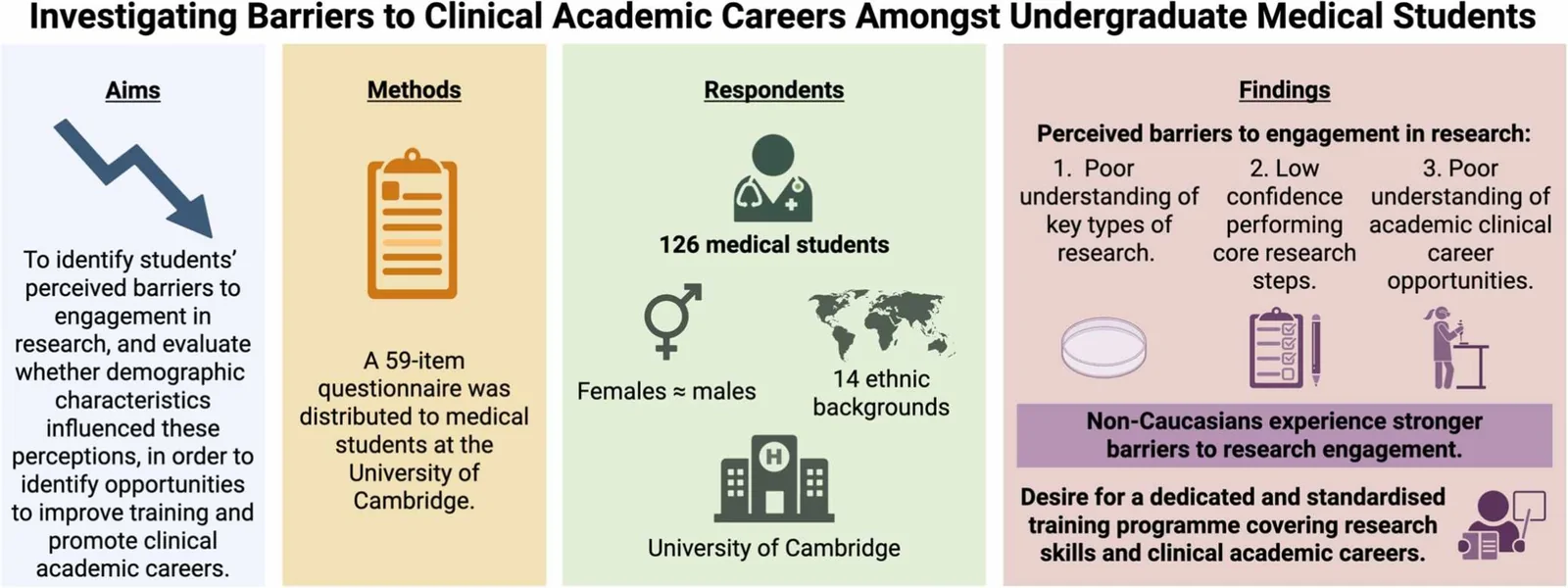
Graphical abstract licence:Created in BioRender. Cole, c. (2026) https://BioRender.com/l5o1sp9*.

## Introduction

There is a critical shortage of academic clinicians in the UK, with the number of academic clinical lecturers falling by 25% between 2010 and 2022.^1^ Potential early consequences on research productivity are apparent, with a 41% decline in the number of industry clinical trials initiated in the UK between 2017 and 2021.^2^

This issue is compounded by a lack of diversity, with women and ethnic minorities constituting only 25% and 13% of UK professors respectively.^1^ Given research questions and study recruitment may be reflected by the backgrounds of the researchers, this may have profound effects on delivering research that benefits the whole population, with subsequent poorer health outcomes in under-represented population groups.^3^, ^4^, ^5^

A likely contributing factor to this crisis is the attitude of university medical students who must form the future academic clinical workforce. Less than 33% of this group are interested in pursuing an academic training pathway, and over 50% report having either no or little exposure to research.^6^ Thus, a vital question arises: what prevents students from engaging in research?

The limited research that exists emphasises practical issues limiting students’ access to research, including time constraints, lack of supportive mentors, and inadequate signposting of opportunities.^7^, ^8^, ^9^, ^10^ However, little emphasis is paid to reasons why students may not be interested in such opportunities in the first place. Similarly, although the lack of representation of minority ethnic groups in clinical academia is apparent,^11^, ^12^, ^13^, ^14^, ^15^ few studies have investigated the causes for this at the medical student level. A study in the USA showed that Black and Hispanic students felt they would have a harder time succeeding in academia, yet it is unclear whether this can be directly extrapolated to the UK.^16^

In the UK, approximately one-third of undergraduate medical students will study for an intercalated degree, though rates vary widely between institutions.^17^ Options to pursue clinical academic training include formal postgraduate training programmes, such as the Specialist Foundation Programme (SFP), Academic Clinical Fellowships (ACFs), and Academic Clinical Lectureships (ACLs).

As the future of the NHS workforce, engaging medical students in research and equipping them with the skills to pursue clinical academic careers is critical. To address this, our study has four main aims:

1. Evaluate medical students’ engagement in research and the extent to which they feel it is sufficient for their future career plans.
2. Identify students’ perceived barriers to engagement in research.
3. Investigate the impact of students’ demographic characteristics on their perceptions of research barriers.
4. Determine students’ perspectives on the potential value of teaching to overcome these barriers.

## Methods

### Survey design and distribution

This study sampled medical students enrolled at the University of Cambridge, at all stages of their medical degree (a potential respondent pool of approximately 1,960 students). The questionnaire was distributed to medical students between November 2023 and January 2024 through a standardised message circulated via medical school mailing lists (reaching all medical students), social media accounts of medical school student societies, and dissemination to medical student groups at the level of the University’s constituent colleges.

A 59-item structured questionnaire [Appendix 1] was created using an online survey platform (Google Forms). This survey was developed through defining the topic of interest and scope of the survey by the Teaching Committee of the Cambridge University Students’ Clinical Research Society, with consensus design using questions based on review of existing literature on the subject and review of academic job application scoring. Survey responses were anonymised. There were no incentives offered for completing the survey. All questions were set to mandatory ensuring incomplete surveys could not be submitted.

The survey was pre-tested prior to full implementation by a trial cohort of students – including student representatives (RVK, AI) and an early career researcher representative (an Academic Clinical Fellow, PRK) – prior to a final review by the Principal Investigator (NRE) to reflect the knowledge, skills, and attitudes covered in the person specifications for clinical academic appointments. The revised survey was then approved by our local institutional review board prior to dissemination to potential responders.

### Survey content

The survey was divided into four sections in line with our key objectives:

1. Baseline characteristics
2. Previous research experience
3. Perceived barriers to research engagement:
  a. Knowledge of research types and skills
  b. Awareness of available research opportunities
  c. Understanding of academic research careers
4. Potential value of research teaching

The ‘perceived barriers to research engagement’ (section three) consisted of 45 questions that used a five-point Likert scale to evaluate students’ awareness, understanding, or confidence relating to types of research, research skills and conduct, and academic pathways. This produced a total (global) score of confidence in research between 45 and 225. We extrapolated that reported reduced awareness, understanding, or confidence relating to these components of research represented a perceived barrier to engaging with research.

Baseline characteristics included student-reported year of the course, sex, and ethnicity.

### Statistical analysis

Data was analysed using R studio (version 4.3.3). Frequency analysis was performed, and the results of the Likert scales were reported as percentages. Spearman’s rank correlation coefficients were used to determine the relationship between different barriers to research engagement and satisfaction with current research teaching.

When considering the impact of the students’ year of training, sex, and ethnicity, the global score of confidence in research was first compared using univariable analysis. Continuous data was tested for normality using the Shapiro-Wilk method, with comparison between groups tested using the Student’s *t*-test (parametric data) or the Wilcoxon Rank Sum Test (non-parametric data). Comparison between multiple groups utilised ANOVA (parametric data) or Kruskal-Wallis (non-parametric) methodologies as appropriate.

We then performed linear regression analysis of the global score of confidence in research considering each of these three demographic criteria. Although considered as separate groups in univariable analysis, for the purposes of the regression analysis, ethnicity was categorised as ‘non-Caucasian’ and ‘Caucasian’ as pre-specified group based on the concern of under-representation in medical research from clinical academics from non-Caucasian backgrounds, consistent with that described in the literature.^18^ Due to an association between year of study and ethnicity, this was included as an interaction term in our model.

### Ethical approval

Using the National Research Ethics Service Decision Tool, it was determined that the study did not require NHS Research Ethics Committee Approval. The study was registered locally with the Cambridge University Hospitals Clinical Audit Registration and Management Service prior to data collection (registration: CARMS-ID5811, PRN11811). Participation was voluntary and respondents did not receive any financial compensation for participation. All participants provided written electronic informed consent for their anonymised data to be used in publication.

## Results

### Demographics

One hundred and twenty-six students responded [Table 1]. Respondents were 45.2% (n = 57) male vs 53.2% (n = 67) female (two students, 1.6%, preferred not to answer). There was representation from 14 ethnic backgrounds, with the largest groups identifying as Chinese (n = 27, 21.4%), White British (n = 24, 19.0%) and Indian (n = 24, 19.0%).

**Table 1:** Baseline demographics of participants (n = 126).

Ethnicity	Number of participants
Chinese	27 (21.4%)
English/Welsh/Scottish/Northern Irish/British	24 (19.0%)
Indian	24 (19.0%)
Any other White background	12 (9.5%)
Any other Asian background	9 (7.1%)
White and Asian	8 (6.3%)
Arab	5 (4.0%)
Pakistani	5 (4.0%)
African	3 (2.4%)
Bangladeshi	3 (2.4%)
Any other Black/African/Caribbean background	2 (1.6%)
Irish	2 (1.6%)
Any other Mixed/Multiple ethnic background	1 (0.8%)
Any other ethnic group	1 (0.8%)
**Sex**	
Female	67 (53.2%)
Male	57 (45.2%)
Prefer not to say	2 (1.6%)
**Year of Study**	
Year 1, Undergraduate Course	45 (35.7%)
Year 2, Undergraduate Course	33 (26.2%)
Year 3, Undergraduate Course	14 (11.1%)
Year 4, Undergraduate Course	25 (19.8%)
Year 5, Undergraduate Course	3 (2.4%)
Year 1, Postgraduate Course	4 (3.2%)
Year 2, Postgraduate Course	1 (0.8%)
Intercalation Year	1 (0.8%)

### Objectives

#### Objective 1: evaluate medical students’ engagement in research and the extent to which they feel it is sufficient for their future career plans

Most students reported limited participation in research: 67.5% (n = 85) had not undertaken a substantial research placement. Furthermore, 93.7% (n = 118) had not authored a publication, and 92.1% (n = 116) had not presented at a national or international conference.

Consequently, whilst most students agree or were neutral that they are interested in applying to the SFP (n = 111, 88.1%), they are not confident that their current research portfolio would be good enough to secure a place on this training pathway (n = 104, 82.5%).

#### Objective 2: identify students’ perceptions of barriers to engagement in research

##### Low confidence in research-related knowledge and skills

Only a minority of respondents report a detailed understanding of the types, purposes, and structure of pre-clinical (n = 56, 44.4%) and clinical (n = 49, 38.9%) research. Furthermore, few report that they are confident in performing key research skills, such as determining a research question, collecting data, performing statistical analyses, and writing an abstract [Fig. 1]. This highlights students’ perceived low confidence in research knowledge and skills that may be preventing them undertaking research.

**Fig. 1:**
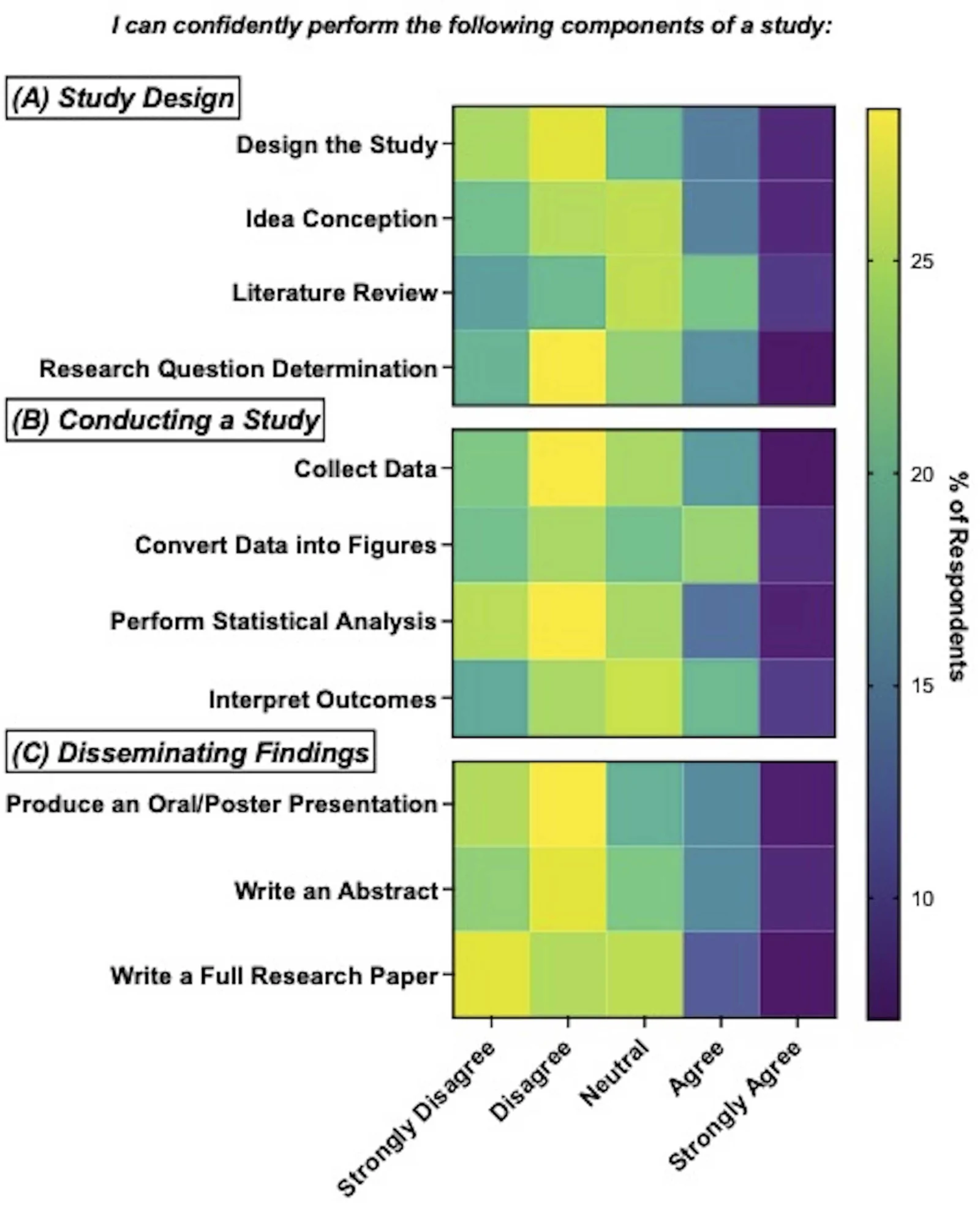
Students’ confidence in performing core research skills (A) before, (B) during and (C) after a study (n = 126); based on a five-point Likert scale, where 1 is strongly disagree and 5 is strongly agree.

##### Lack of awareness of opportunities to get involved in research

Across the different types of research, there was a moderate correlation between the students’ level of knowledge about a type of research and their awareness of opportunities to get involved in that type of research [Supplementary material]. In particular, only a minority reported feeling some confidence or very confident in finding opportunities to conduct clinical studies (n = 26, 20.6%) and systematic or narrative reviews (n = 23, 18.3%).

Few students are confident contacting a researcher to seek research opportunities (n = 36, 28.6%) and knowing where to find grants or funding (n = 17, 13.5%) for a project. Only a small proportion of students are aware of opportunities to disseminate their research through conferences (n = 13, 10.3%), journals (n = 15, 11.9%), or prizes (n = 15, 11.9%).

##### Insufficient understanding of academic research careers

Most students were unaware of the SFP [Fig. 2]. This awareness is higher in those in latter stages of their medical school training (median score on a 5-point Likert scale 3 (IQR:2.00) in pre-clinical students vs 5 (IQR:1.25) in clinical students, p < 0.001). However, even then 25.0% (n = 7) of clinical students gave a score of 3 or less. Furthermore, the structure of clinical academic training and careers beyond SFP, such as Academic Clinical Fellowships or Senior Lecturer positions, is poorly understood by all medical students [Fig. 2].

**Fig. 2:**
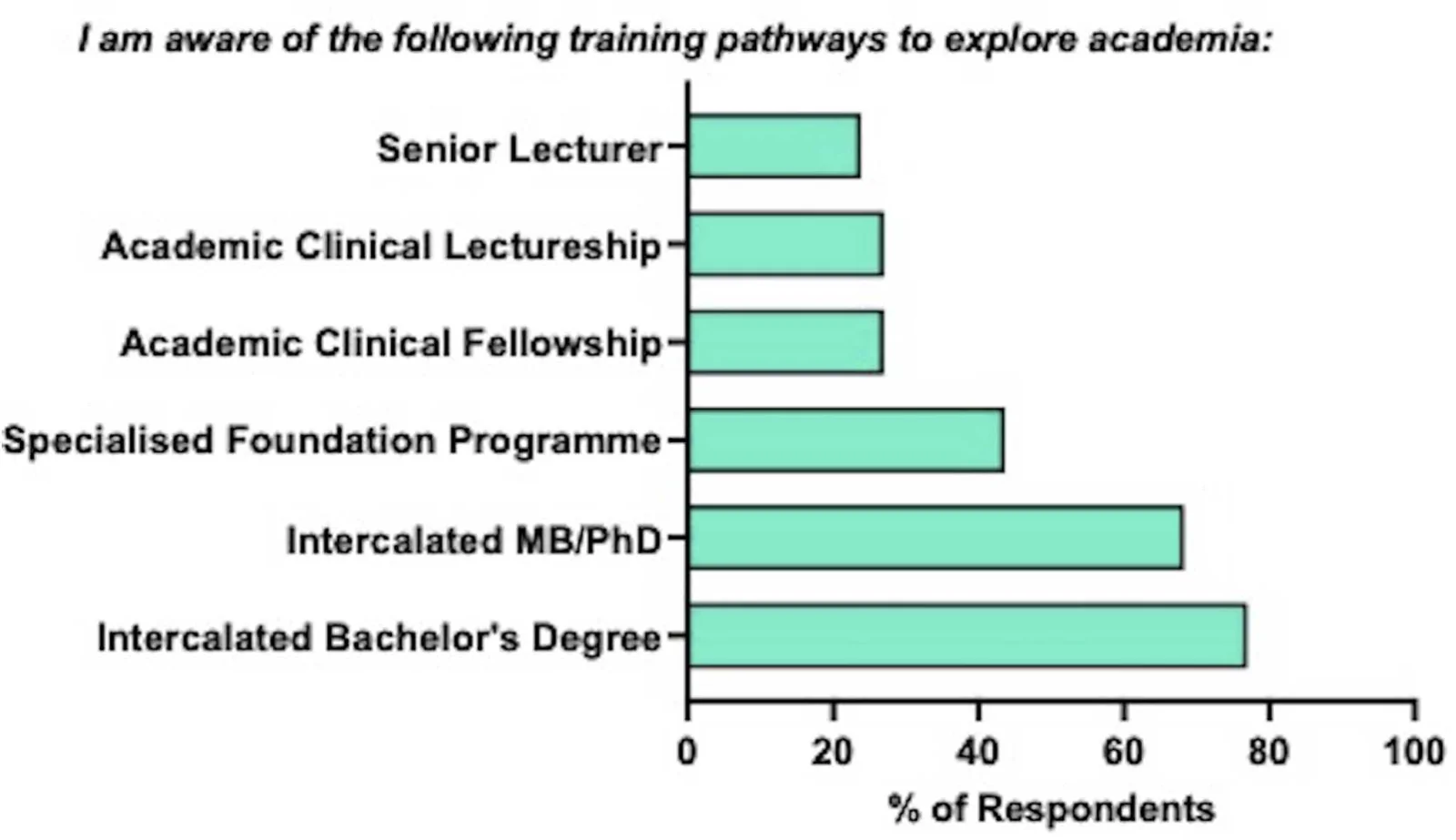
Students’ awareness of opportunities to explore academia (n = 126).

#### Objective 3: investigate the impact of students’ demographic characteristics on their perceptions of research barriers

On univariable analysis, there was no significant difference between the mean global score of confidence in research between male and female medical students (108.2 ± 36.3 versus 113.1 ± 32.9, p = 0.43).

When considering year of study, there was a mild positive association with the global score of confidence in research (r_s_ = 0.28, p < 0.01).

On Kruskal-Wallis testing, there was no significant difference in median global score of confidence in research between different ethnic groups (p = 0.48). There was also no significant difference in the mean global score of confidence in research between students from Caucasian and non-Caucasian backgrounds (116.9 ± 33.0 versus 108.5 ± 34.9 respectively, p = 0.21).

Considering these factors in a linear regression analysis demonstrated each year of study was independently associated with a 12.46-point increase in the global score of confidence in research (equivalent to 5.5% of the total of the maximum score available). In contrast, non-Caucasian ethnicity was independently associated with a 26.36-point lower score than for Caucasian ethnicity (equivalent to 11.7% of the maximum available score). Sex was not independently associated with the global score of confidence in research after multivariable adjustment (Table 2).

**Table 2:** Linear regression modelling for the global score of confidence in research.

	Beta	Significance
Year of study	12.46	P < 0.001
Non-Caucasian ethnicity	−26.36	P = 0.047
Male sex	−0.92	P = 0.88

#### Objective 4: determine students’ perspectives on the potential value of a dedicated training programme to overcome these barriers

Most students are dissatisfied with the amount of teaching received on research portfolio development (n = 108, 85.7%). Students with a lower global score of confidence are more dissatisfied with the amount of teaching obtained on research portfolio development (ρ = 0.51, p < 0.001). The majority of students (n = 99, 78.6%) supported that further research teaching would be beneficial for them, suggesting it may help overcome these challenges.

## Discussion

Our study demonstrates^1^: medical students demonstrate low levels of experience and exposure relating to medical research^2^; insufficient research skills, opportunities and understanding of academic careers are likely barriers preventing greater engagement^3^; perceptions of these barriers are heightened in those of non-Caucasian ethnicity; and^4^ there is a high level of demand amongst students for a dedicated and standardised training programme covering research skills and clinical academic careers.

### Barriers to engagement in research: skills, opportunities and understanding

Students indicate low levels of understanding of pre-clinical and clinical research, a lack of confidence in performing essential research skills, and low awareness of academic career opportunities as barriers to engagement in research. Whilst studies usually focus on a single class of obstacle,^6^, ^16^, ^19^, ^20^, ^21^, ^22^, ^23^ a strength of this study is the categorisation of multiple known barriers to research engagement into actionable domains.

Firstly, it is subjective confidence in research skills, rather than objective attainment, that is a stronger predictor of pursuing an academic career.^24^, ^25^ Therefore, the fact that fewer than one in three students were confident in performing basic research steps is concerning and can be corroborated by low confidence scores in other studies.^26^, ^27^

Secondly, only one in five students were confident finding routes to engage in research. Previous work has focused on practical barriers – such as time constraints, funding challenges, and infrequent networking^28^ – as a cause for this. However, our findings show that students also suffer from a lack of opportunity to disseminate their work and engage with the wider research community. This may generate a perceived barrier that discourages future research involvement, especially as portfolio development is often a key motivator for conducting research.^29^

Thirdly, we highlight the lack of awareness of clinical academic training structures. Students cannot develop an interest in a training pathway about which they are unaware.^30^ Students in previous focus groups described the process for pursuing academic careers as ʻsecretive’.^16^ We confirm this in a larger cohort by showing that more than half of students are unaware of the stages of an academic pathway post-university. Raising awareness of such training programmes and demystifying their selection and structure is vital.

### Ethnic disparities in barriers to research engagement

Similar barriers to research engagement – including a lack of opportunities, a lack of prior research opportunities, and a lack of confidence in performing common academic tasks – have been reported in a similar single-centre UK study, and our study adds valuable complementary results that highlight the impact of ethnicity.^31^ This subgroup analysis suggests that non-Caucasian medical students perceive greater barriers to their involvement in research compared to their Caucasian peers. This finding is consistent with other studies that identify racial biases within academic medicine in smaller focus groups or in other countries.^16^, ^19^, ^20^ Furthermore, although Parameswaran *et al*. found that Asian students were more likely to want to pursue an academic pathway,^6^ only 10.4% of UK Professors are Asian, compared to White Professors who constitute 66.2% of the workforce.^32^ This is consistent with the finding that the clinical academic workforce (particularly more senior positions) is less diverse than the wider medical or healthcare workforce.^18^, ^20^, ^33^, ^34^, ^35^ Our findings suggest that minority ethnic students have lower confidence in their research skills, their ability to find research projects, and their understanding of the academic career pathway, all of which may contribute to a sense of disadvantage for these students. The important implications from these findings serve as an important proof of principle and warrant corroboration in larger studies with sufficient statistical power to differentiate between different ethnicities and to accommodate a broader range of potential confounders.

To aid expansion and diversification of the dwindling academic workforce, we advocate for inclusive research training early in medical school curricula to ensure equality, diversity and inclusion are a priority early in the academic career pathway.^36^, ^37^, ^38^ Specifically, multiple studies highlight the importance of suitable mentors to conduct this teaching,^39^, ^40^ and that they should reflect the entire population they teach. Minority ethnic role models have been shown to be the most significant facilitator for diverse students entering academia.^19^, ^20^ Furthermore, all mentors, regardless of background, should seek to build meaningful mentor-mentee relationships with minority ethnic students. Only a quarter of students felt confident in contacting a researcher; therefore, a teaching programme should facilitate interactions between students and potential supervisors in order to foster the community of practice and minimise barriers related to perceived approachability of clinical academics.

### Standardised training in medical research is a priority

Only 19.6% of students globally considered research was sufficiently addressed in core medical school curricula,^41^ comparable to the findings in our UK-based cohort. In addition, our results demonstrate that students who are more dissatisfied with research teaching have a lower global score of confidence in research, and hence experience stronger barriers towards engaging in research. Differential geographical attainment has been noted within the SFP,^42^ which has the potential to extend into the remainder of the academic pathway, and hence the identified need for dedicated research training may be best addressed through a national standardised programme of research training activities in order to address these educational inequalities. To guide development of a targeted educational intervention, this study brings to light specific gaps in research training in undergraduate education.

### Limitations

The term ʻNon-Caucasians’ represents a large number of ethnicities and it would be useful to understand differences in perceived barriers between ethnicities within this group. Although comparison between groups did not demonstrate significant differences, there were not enough respondents of certain ethnicities to make firm conclusions, and so we categorised students in line with previous publications that compared White to Black and Minority Ethnic cohorts, such as the NHS Medical Workforce Race and Equality Standard.^43^ This acts as a pilot study for larger studies with sufficient power to detect specific differences. This will enable greater targeting of interventions to the ethnicities most affected by the barriers identified in this study. Furthermore, a larger study may facilitate consideration of other potential confounders, such as socioeconomic status, which was not considered in our study.

We acknowledge a risk of participation bias, judging by the high level of interest in research indicated by our cohort. It is likely that students who are already interested in research were more likely to respond to the survey, which may overestimate views on dissatisfaction with research teaching.

Our study presents the data from a single institution, though supports and expands on trends seen in other UK centres.^31^ Our institution may not be typical of other institutions – where the local curriculum, student make-up, or general learning environment may act as potential confounders – and hence there is the potential for bias that may either over- or under-estimate the research barriers encountered at a national level. However, there remains under-representation from some institutions and regions in the wider literature, and hence further work with a larger cohort from across the country is essential to understand how these findings relate to the wider UK medical student body.

Nonetheless, future study on the impact of the newly introduced preference-informed allocation of the SFP is necessary. Although it may be that reducing application points relating to research experience may disincentivise participation, and consequently reduce interest, in research,^44^ it may also have the effect of democratising participation in research through a system that equally awards students regardless of prior research engagement, thereby potentially attenuating the impact of the barriers to research engagement identified in our study.

## Conclusion

Overall, students reported low levels of understanding of pre-clinical and clinical research, a lack of confidence in performing essential research skills, and low awareness of academic career opportunities as barriers to engagement in research. These barriers to participating in research were more common in those of non-Caucasian ethnicity. Efforts to promote an academic workforce that reflects the wider healthcare organisation and general population are necessary to improve access for ethnic minorities, ensuring equality, diversity and inclusion at all stages of the clinical academic and research pathways.

## CRediT authorship contribution statement

**Ranya V. Kumar:** Writing – review & editing, Writing – original draft, Visualization, Validation, Supervision, Software, Resources, Project administration, Methodology, Investigation, Funding acquisition, Formal analysis, Data curation, Conceptualization. **Caleb Cole:** Writing – review & editing, Writing – original draft, Visualization, Validation, Supervision, Software, Resources, Project administration, Methodology, Investigation, Funding acquisition, Formal analysis, Data curation, Conceptualization. **Jacob Vincent:** Writing – review & editing, Writing – original draft, Visualization, Validation, Supervision, Software, Resources, Project administration, Methodology, Investigation, Funding acquisition, Formal analysis, Data curation, Conceptualization. **Prakrit R. Kumar:** Writing – review & editing, Writing – original draft, Visualization, Validation, Supervision, Software, Resources, Project administration, Methodology, Investigation, Funding acquisition, Formal analysis, Data curation, Conceptualization. **Nicholas R. Evans:** Writing – review & editing, Writing – original draft, Visualization, Validation, Supervision, Software, Resources, Project administration, Methodology, Investigation, Funding acquisition, Formal analysis, Data curation, Conceptualization.

## Ethics approval and consent to participate

Using the National Research Ethics Service Decision Tool, it was determined that the study did not require NHS Research Ethics Committee Approval. The study was registered locally with the Cambridge University Hospitals Clinical Audit Registration and Management Service (CARMS) (registration ID: CARMS-ID5811, PRN11811) prior to data collection. Participation was voluntary and respondents did not receive any financial compensation for participation. All participants provided written electronic informed consent for their anonymised data to be used in publication.

## Funding

This work has been supported by the INSPIRE Programme Grant from the 10.13039/501100000691Academy of Medical Sciences. NRE is supported by a Stroke Association Senior Clinical Lectureship [SA-SCL-MED-22\100006] and by the NIHR Cambridge Biomedical Research Centre (NIHR203312). The views expressed are those of the authors and not necessarily those of the NIHR or the Department of Health and Social Care.

## Declaration of competing interest

The authors declare the following financial interests/personal relationships which may be considered as potential competing interests: Nicholas Evans reports financial support was provided by INSPIRE Programme Grant from the Academy of Medical Sciences (IR6). If there are other authors, they declare that they have no known competing financial interests or personal relationships that could have appeared to influence the work reported in this paper.

## Data Availability

The data that support the findings of this study are available from the corresponding author upon reasonable request.

## Footnotes

Appendix A Supplementary data associated with this article can be found in the online version at doi:10.1016/j.clinme.2026.100657.
