# Supplementary material for: Investigating barriers to clinical academic careers amongst undergraduate medical students
Source: Clin Med (Lond). 2026 Oct 18;26(6):100657. doi: 10.1016/j.clinme.2026.100657 (PMC13737729; doi:10.1016/j.clinme.2026.100657)
Supplement: Supplementary file 1 [file mmc1.pdf]

Evaluating Medical Research Education Amongst Medical Students

Thank you for taking the time to complete this survey. The aim of this study is to assess the current research experience of medical students in the year 2023-24. This study is led by Cambridge University Students' Clinical Research Society, an organisation which wishes to address the gap in research teaching in Medical schools in the United Kingdom. The survey consists of only multiple choice questions and should take approx. 5 minutes to complete.

If you have any queries or would like further information please contact: cuscrs@gmail.com

Confidentiality Statement: CUSCRS is a not-for-profit organisation which maintains strict confidentiality requirements and regulations in accordance with the Data Protection Act 2018. All responses will be anonymised during the process of data collection and analysis. No person identifiable data will be collected during this audit process.

*Required

* Indicates required question

1. Do you consent to participate in this study? *

Mark only one oval.

- ( ) Yes
- ( ) No

2. Year of study *

Dropdc

Mark only one oval.

- Year 1, Undergraduate Course
- Year 2, Undergraduate Course
- Year 3, Undergraduate Course
- Year 4, Undergraduate Course
- Year 5, Undergraduate Course
- Year 1, Postgraduate Course
- Year 2, Postgraduate Course
- Year 3, Postgraduate Course
- Year 4, Postgraduate Course
- Intercalation Year
- FY1
- FY2
- Other

3. University of study *

Dropdc

Mark only one oval.

- University of Aberdeen School of Medicine and Dentistry
- Anglia Ruskin University School of Medicine
- Aston University Medical School
- Queen Mary University of London
- University of Birmingham College of Medical and Dental Sciences
- Brighton and Sussex Medical School
- University of Bristol Medical School
- University of Buckingham Medical School
- University of Cambridge School of Clinical Medicine
- Cardiff University School of Medicine
- University of Dundee School of Medicine
- Edge Hill University Medical School
- The University of Edinburgh Medical School
- University of Exeter Medical School
- University of Glasgow School of Medicine
- Hull York Medical School
- Imperial College London Faculty of Medicine
- Keele University School of Medicine
- Kent and Medway Medical School
- King's College London GKT School of Medical Education

- Lancaster University Medical School
- University of Leeds School of Medicine
- University of Leicester Medical School
- University of Liverpool School of Medicine
- London School of Hygiene & Tropical Medicine
- University of Manchester Medical School
- Newcastle University School of Medical Education
- University of East Anglia, Norwich Medical School
- University of Nottingham School of Medicine
- University of Nottingham - Lincoln Medical School
- University of Oxford Medical Sciences Division
- Plymouth University Peninsula Schools of Medicine and Dentistry
- Queen's University Belfast School of Medicine
- University of Sheffield Medical School
- University of Southampton School of Medicine
- University of St Andrews School of Medicine
- St George's, University of London
- University of Sunderland School of Medicine
- Swansea University Medical School
- University of Central Lancashire School of Medicine

- University College London Medical School
- University of Warwick Medical School
- Brunel University London, Brunel Medical School
- Ulster University, School of Medicine
- University of Chester Medical School
- Three Counties Medical School
- Other (e.g. Non-Medic)

4. Gender *

Mark only one oval.

- Male
- Female
- Other
- Prefer not to say

5. Ethnicity * Dropdc

Mark only one oval.

- English / Welsh / Scottish / Northern Irish / British
- Irish
- Gypsy or Irish Traveller
- Any other White background
- White and Black Caribbean
- White and Black African
- White and Asian
- Any other Mixed / Multiple ethnic background
- Indian
- Pakistani
- Bangladeshi
- Chinese
- Any other Asian background
- African
- Caribbean
- Any other Black / African / Caribbean background
- Arab
- Any other ethnic group

Your Previous Research Experience

6. Number of research placements of 4+ weeks undertaken *

Mark only one oval.

- 0
- 1
- 2
- >2

7. Number of PubMed-cited publications as 1st author or co-author (not collaborative author) *

Mark only one oval.

- 0
- 1
- 2-5
- >5

8. Number of presentations (oral/poster) at national/international conferences *

Mark only one oval.

- 0
- 1
- 2-5
- >5

9. Number of audits or quality improvement projects participated in *

Mark only one oval.

- 0
- 1
- 2-5
- >5

10. Number of prizes or funding grants awarded at a national level *

Mark only one oval.

- 0
- 1
- 2-5
- >5

Your Understanding of a Career in Research

11. I am aware of the following opportunities to explore academia in the medical training pathway: *

Mark only one oval per row.

	Strongly disagree	Disagree	Neutral	Agree	Strongly agree
**Intercalated BA/BSc**	◯	◯	◯	◯	◯
**Intercalated MB/PhD**	◯	◯	◯	◯	◯
**Specialised Foundation Programme (SFP)**	◯	◯	◯	◯	◯
**Non-SFP opportunities in F1/F2**	◯	◯	◯	◯	◯
**Academic Clinical Fellowship/MD-PhD Training Fellowship/Clinical Scientist/Clinical Lectureship**	◯	◯	◯	◯	◯
**Senior Clinical Fellowship/Further sub/speciality training/GP in-practice Fellowship/Senior Lecturer**	◯	◯	◯	◯	◯

12. I fully understand the selection criteria and application process for competitive posts including SFP, ACF, CST and IMT, and I understar how participating in research whilst as medical school would benefit me when applying to these

Mark only one oval.

	1	2	3	4	5	
Stro	◯	◯	◯	◯	◯	Strongly agree

Your Knowledge of Types of Research

13. I fully understand the types, purposes and structure of: *

Mark only one oval per row.

	Strongly disagree	Disagree	Neutral	Agree	Strongly agree
**Pre-clinical research (e.g. wet-lab, dry-lab)**	⬭	⬭	⬭	⬭	⬭
**Clinical research (e.g. RCT, case control, case cohort, cross-sectional)**	⬭	⬭	⬭	⬭	⬭
**Reviews (e.g. systematic reviews, narrative reviews)**	⬭	⬭	⬭	⬭	⬭
**Audits and quality improvement projects**	⬭	⬭	⬭	⬭	⬭
**Medical education research (e.g. teaching programme evaluation)**	⬭	⬭	⬭	⬭	⬭
**Public health research**	⬭	⬭	⬭	⬭	⬭

Your Understanding of the Research Process

14. I can confidently perform the following components of a study: *

Mark only one oval per row.

	Strongly disagree	Disagree	Neutral	Agree	Strongly agree

Idea conception	◯	◯	◯	◯	◯
Literature review	◯	◯	◯	◯	◯
Research question determination	◯	◯	◯	◯	◯
Secure funding	◯	◯	◯	◯	◯
Obtain ethics approval	◯	◯	◯	◯	◯
Design the study	◯	◯	◯	◯	◯
Collect data	◯	◯	◯	◯	◯
Perform statistical analyses of my data	◯	◯	◯	◯	◯
Interpret outcomes (e.g. p-values, confidence intervals)	◯	◯	◯	◯	◯
Convert data into figures	◯	◯	◯	◯	◯
Write the full research paper	◯	◯	◯	◯	◯
Write an abstract to apply to a conference	◯	◯	◯	◯	◯
Produce an oral/poster presentation for a conference	◯	◯	◯	◯	◯

Your Research Opportunities

15. I am aware of possible routes for getting involved in different types of research whilst at medical school, including: *

Mark only one oval per row.

	Strongly disagree	Disagree	Neutral	Agree	Strongly agree
**Pre-clinical/lab-based studies**					
**Clinical studies**					
**Systematic and narrative reviews**					
**Audits and quality improvement projects**					
**Medical education research**					
**Public health research**					

16. I would feel confident contacting researchers regarding completing a research project with them *

Mark only one oval.

	1	2	3	4	5	
Stro	◯	◯	◯	◯	◯	Strongly agree

17. I would know where to look for grants/funding if I wanted to complete a research project *

Mark only one oval.

	1	2	3	4	5	
Stro	◯	◯	◯	◯	◯	Strongly agree

18. I know journals specific to each type of research and which are likely to publish my work (e.g. student BMJ, New Doctor, Junior Doctor

Mark only one oval.

	1	2	3	4	5	
Stro	◯	◯	◯	◯	◯	Strongly agree

19. I know conferences specific to each type of research at which I can present (e.g. ASIT) *

Mark only one oval.

	1	2	3	4	5	
Stro	◯	◯	◯	◯	◯	Strongly agree

20. I know specialty-specific prizes which I could apply for *

Mark only one oval.

	1	2	3	4	5	
Stro	◯	◯	◯	◯	◯	Strongly agree

21. I know medical student-specific prizes which I could apply for *

Mark only one oval.

	1	2	3	4	5	
Stro	◯	◯	◯	◯	◯	Strongly agree

Your Future Career Plans

22. I am interested in applying to the Specialised Foundation Programme *

Mark only one oval.

	1	2	3	4	5	
Stro	◯	◯	◯	◯	◯	Strongly agree

23. I understand the Specialised Foundation Programme application process *

Mark only one oval.

	1	2	3	4	5	
Stro	◯	◯	◯	◯	◯	Strongly agree

24. I understand the Specialised Foundation Programme shortlisting criteria *

Mark only one oval.

	1	2	3	4	5	
Stro	◯	◯	◯	◯	◯	Strongly agree

25. I am confident that my current research portfolio is good enough to secure the Specialised Foundation Programme and/or an academic career

Mark only one oval.

	1	2	3	4	5	
Stro	◯	◯	◯	◯	◯	Strongly agree

26. I understand how to improve my research portfolio in preparation for applying for Specialised Foundation Programme *

Mark only one oval.

	1	2	3	4	5	
Stro	◯	◯	◯	◯	◯	Strongly agree

What Education Would Benefit You?

27. Number of hours of teaching offered on how to build a research portfolio as part of core medical school curriculum *

Mark only one oval.

- ◯ 0
- ◯ 1-2
- ◯ 3-10
- ◯ >10

28. I feel satisfied with the amount of teaching I have received on how to build a research portfolio *

Mark only one oval.

	1	2	3	4	5	
Stro	◯	◯	◯	◯	◯	Strongly agree

29. I know where to find teaching on research skills that is medical student-tailored *

Mark only one oval.

	1	2	3	4	5	
Stro	◯	◯	◯	◯	◯	Strongly agree

30. I would benefit from a teaching programme on how to build my research portfolio to assist with applications for: *

Mark only one oval per row.

	Strongly disagree	Disagree	Neutral	Agree	Strongly agree
**Future academic career (e.g. Specialised Foundation Programme)**	⬭	⬭	⬭	⬭	⬭
**Competitive clinical career (e.g. Core Surgical Training)**	⬭	⬭	⬭	⬭	⬭
**Posts in competitive deaneries**	⬭	⬭	⬭	⬭	⬭

This content is neither created nor endorsed by Google.

Google Forms
