# Supplementary material for: Investigating barriers to clinical academic careers amongst undergraduate medical students
Source: Clin Med (Lond). 2026 Oct 18;26(6):100657. doi: 10.1016/j.clinme.2026.100657 (PMC13737729; doi:10.1016/j.clinme.2026.100657)
Supplement: Supplementary file 2 [file mmc2.docx]

| Table A: Correlations between students’ knowledge of types of research and their awareness of opportunities to pursue research within the corresponding type of research (n=126) | | |
| --- | --- | --- |
|  | ***ρ*** | **p value** |
| Clinical Research | 0.5086 | <0.0001 |
| Preclinical Research | 0.6204 | <0.0001 |
| Systematic and Narrative Reviews | 0.5518 | <0.0001 |
| Medical Education Research | 0.5966 | <0.0001 |
| Public Health Research | 0.6239 | <0.0001 |
